# Should compulsory admission to hospital be part of suicide prevention strategies?

**DOI:** 10.1192/pb.bp.116.055699

**Published:** 2017-06

**Authors:** Daniel Wei L. Wang, Erminia Colucci

**Affiliations:** 1 School of Law, Queen Mary University of London; 2 Wolfson Institute, Queen Mary University of London

## Abstract

The World Health Organization report *Preventing Suicide: A Global Imperative* provides governments with guidance for comprehensive suicide prevention strategies. However, it does not mention the role that compulsory admission to hospital of psychiatric patients should have in policies for suicide prevention. This was a missed opportunity for international discussion and guidance about a measure that, although widely used, is becoming increasingly controversial in light of the existing evidence and human rights norms.

Legal and policy approaches to suicide have been changing significantly in the past decades. Notably, the decriminalisation of suicide attempts in many countries has been followed by an increasing recognition that the stigma around suicide must be grappled with and that adequate social and healthcare policies can reduce the rate of suicide in the population. In other words, the focus has shifted from criminal punishment and moral condemnation to awareness, support and prevention.^1^

The 2014 World Health Organization (WHO) report *Preventing Suicide: A Global Imperative*^1^ is an important step in this direction. It draws attention to the discrepancy between the magnitude of suicide as a health problem worldwide and the low priority it is given by national governments. The report also describes the risk and protective factors in suicide prevention based on state of the art research and offers guidance for comprehensive strategies for suicide prevention. The central message in the document is that suicides are preventable but this requires actions such as restricting access to the means of suicide, reducing excessive use of alcohol, collecting and collating good-quality data about suicide and suicide attempts, providing training for gatekeepers, improving the quality of mental healthcare services, and promoting responsible reporting of suicide by the media. The report is also clear that taboo, stigma, shame, guilt and discrimination surrounding suicide hamper effective suicide prevention policies as they discourage vulnerable people from seeking help.

However, despite its emphasis on the need for comprehensive strategies for suicide prevention, the compulsory admission to hospital of people at risk of suicide was not discussed in the WHO report. This should not come completely as a surprise given that compulsory admission to hospital was also ignored in the previous United Nations (UN) and WHO documents on which this report was built.^2–4^ Moreover, the literature on suicide prevention rarely lists compulsory admission to hospital among the measures for suicide prevention, and those who do normally do not distinguish between voluntary and compulsory admission.^5–7^

This gap in international guidelines and in the scholarly literature needs to be addressed. Compulsory admission to hospital is widely used as a measure for suicide prevention, but the trade-offs involved and the human rights implications make it a topic in which guidance and further discussion are urgently needed.

## Compulsory admission to hospital for suicide prevention

Compulsory admission to psychiatric hospitals or psychiatric wards is allowed in many countries as a measure to prevent self-harm.^8^ In England and Wales, for instance, the Mental Health Act 1983 (MHA) provides the legal framework for the compulsory admission and treatment of patients with mental disorders of a nature or degree that warrants their detention in a hospital and who ought to be so detained in the interests of their own health or safety or with a view to the protection of other persons. Whether the patient has capacity to decide on their stay in hospital and has objected to it will not affect the legality of a detention under the MHA. A recent publication shows that there were over 63 000 detentions under the MHA in the period from 1 April 2015 to 31 March 2016.^9^ Considering the body of literature associating suicide with mental disorders^6,10^ (see, however, Hjelmeland *et al*^11^) and that statistically people with mental disorders are at a higher risk to themselves than to others,^12^ it is plausible to assume that prevention of self-harm is a common reason for compulsory admission to hospital.

Some would interpret this authorisation to detain as actually a duty to detain when there is a high and immediate risk of a person taking their own life. A failure to do so can be considered medical negligence and may also be a breach of human rights. In the case of *Rabone & Anor v Pennine Care NHS Foundation Trust* [2012],^13^ the Supreme Court unanimously held that the failure of the hospital staff to detain Melanie, a voluntary psychiatric patient who hanged herself from a tree after being allowed to spend the weekend with her family, was a breach of her right to life under Article 2 of the European Convention on Human Rights. According to the Court, given her history of depression and self-harm, including a previous suicide attempt, the hospital staff should have used their powers to detain Melanie under the MHA to protect her from the ‘real and immediate risk of suicide’ when she demanded to leave the hospital.

Even though this precedent applies to the UK only, it shows that a national strategy for suicide prevention may be incomplete without a policy for compulsory admission to hospital. In hindsight, it is clear that the deaths of people like Melanie could have been avoided were they admitted to hospital and put under close observation, treated, managed and prevented from having access to the means to take their own life.

## Compulsory admission to hospital: trade-offs and human rights

When looking at individual cases of suicide and at the data from population-based studies there is evidence that compulsory admission to hospital saves lives.^14,15^ However, this does not answer the question of how, when or whether it should be used to prevent suicide. Compulsory admission to hospital involves trade-offs and has human rights implications that need to be considered in clinical, policy and legal decisions about its role in strategies for the prevention of suicide.

There is now compelling evidence that suicide, being a low-frequency event, is very difficult to predict. The clinical methods for predicting suicide among patients have a very poor predictive capacity.^16–20^ A recent meta-analysis revealed that, over an average follow-up of 5 years, almost half of all suicides are likely to occur in patients considered at low risk, and that 95% of high-risk patients will not die by suicide.^21^ This creates a trade-off between the need to be sensitive to the risk of suicide to reduce the chance of false negatives and the need to be specific to avoid false positives that may lead to unnecessary detentions. Assuming that it is impossible to predict whether a person is going to take their own life and that the best we can do is to estimate that 1 out of *X* people in a certain cohort will die by suicide, then a society that allows compulsory detention of people at risk of suicide has to admit that to save one person it will have to unnecessarily detain (*X* − 1) people.

There are also concerns about whether compulsory detention may increase the risk of suicide in some cases. First, some people may not seek treatment because they are fearful of being forced to accept treatments not of their choice or of being detained for prolonged periods.^22^ This would go against the WHO recommendation that a national effort to prevent suicide should encourage people to seek help. Second, there is an association between suicide and psychiatric admission to hospital, as suicide risk peaks in the period immediately after admission to hospital and shortly after discharge.^5,6,14,23,24^ This association can be explained in part by the fact that individuals with higher risk of suicide are more likely to be admitted to hospital,^25^ but some argue that admission to psychiatric in-patient care might actually increase the risk of suicide. The stigma, discrimination, impact on employability, trauma, isolation and the feeling of dehumanisation caused or augmented by compulsory admission to hospital may contribute to the extremely high risk of suicide in the first few days of admission and after discharge.^17,22,26,27^ Although further research is necessary, this hypothesis does not seem farfetched given that people who are detained, disconnected from their social circle and experience trauma, abuse and emotional distress are at a higher risk of suicide.^1^ Hence, it is possible that some of the (*X*−1) people unnecessarily detained will in fact die by suicide as a result of compulsory admission.

The trade-offs and tragic choices in compulsory admission to hospital have clear human rights implications. Health systems and professionals who are under pressure to be sensitive to the risk of suicide to avoid breaching a patient's right to life will do so at the expense of specificity. This leads to an increase in unnecessary detentions, which interferes with the right to freedom of movement, autonomy, bodily integrity and private life of those detained. It may also affect the right to life of those whose risk of suicide increased as a result of their stay in hospital. Therefore, the rules and practices regarding the compulsory admission to hospital of people with mental disorders to prevent suicide are always choices between different rights and rights-holders.

There are also concerns about whether compulsory admission to hospital is inherently discriminatory against people with mental disorders as it denies them the right to decide about their own treatment. This concern is reflected in the discussions about whether compulsory admission to hospital is compatible with the UN Convention on the Rights of Persons with Disabilities (CRPD), in particular Article 14, which establishes that ‘the existence of a disability shall in no case justify a deprivation of liberty’. The UN Committee on the Rights of Persons with Disabilities,^28^ for instance, affirms in its guidelines on Article 14 of the CRPD that the ‘legislation of several States parties, including mental health laws, still provide instances in which persons may be detained on the grounds of their actual or perceived impairment, provided there are other reasons for their detention, including that they are deemed dangerous to themselves or others. This practice is incompatible with Article 14 […]’. Others, however, worry about how the prohibition of compulsory detention and treatment for people with mental disorders will affect the protection of other rights of people with disabilities, such as their rights to health and to life.^29^

## The need for guidance

In conclusion, four things can be said about compulsory admission to hospital as a measure for suicide prevention. First, it can save the lives of those who, without the care, treatment and management received in hospital, would have taken their own life. Second, owing to the poor suicide predictive capacity of the existing methods, false positives will occur and this results in unnecessary hospital admissions, which can be aggravated if legal accountability encourages defensive clinical practice. Third, there is the possibility that compulsory admission to hospital is partially responsible for the suicides of those who failed to seek help owing to the fear of involuntary detention or for whom the experience of being admitted to hospital contributed to the decision to take their own life. Fourth, it is still unclear how and if compulsory admission to hospital of people on the basis of their mental impairment and the risk of danger to themselves can be reconciled with the CRPD.

The trade-offs involved and the need for measures for the prevention of suicide to be compliant with human rights make the creation of guidelines concerning their use challenging, but necessary. The WHO is a forum in which an evidence-informed, international, multi-stakeholder discussion can shed light on the role (if any) that compulsory admission to hospital should have in a national policy for the prevention of suicide. It is unfortunate that the otherwise commendable 2014 report missed this opportunity. It may be uncomfortable for those advocating policies to prevent suicide to discuss compulsory admission to hospital as this is a measure in which the line that separates protection and harm can be very thin, and there is controversy about where it lies. However, as those working in the area of suicide prevention already know, avoiding a difficult issue is never the best way to deal with it.
